# Molecular response prediction in CML: novel ideas?

**DOI:** 10.18632/oncotarget.21049

**Published:** 2017-09-19

**Authors:** Dominik Wolf, Sieghart Sopper

**Affiliations:** Department of Hematology, Oncology and Rheumatology, Center of Integrated Oncology Cologne Bonn, University Hospital of Bonn, Bonn, Germany

**Keywords:** CML, TKI

**Figure 1:**
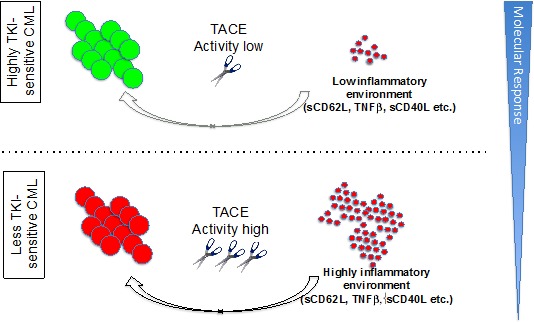
Proposed involvement of TACE in CML biology

Since the introduction of tyrosine kinase inhibitor imatinib, the first targeted cancer therapy, survival of Chronic Myelogenous Leukemia (CML) patients has approached that of the normal population [1]. Treatment with TKI leads to reduction of malignant cells by several orders of magnitude, sometimes even below the level of detection of very sensitive molecular assays. Thus, in recent years achievement of a continuous reduction by more than 4 log, called deep molecular response (DMR), which prevents disease progression and allows for tyrosine kinase inhibitor (TKI) therapy discontinuation (i.e. treatment-free remission=TFR) in patients suffering from CML has emerged as main treatment goal. Second generation TKIs (e.g. nilotinib, dasatinib and bosutinib) induce a higher probability to achieve this landmark when compared to the first generation TKI imatinib [2]. Novel non-ATP competitive BCR-ABL inhibitors, such as ABL001 are emerging tools that may further improve DMR rates. Interestingly, TFR upon TKI withdrawal is linked to several clinical variables, including duration of previous TKI therapy, time of previous deep molecular remission and clinical risk scores (i.e. SOKAL). Of note, recent evidence suggests that immunological biomarkers at the time of TKI discontinuation are associated with the likelihood of continuous TFR, including increased NK cells [3] and CD86 positive pDC [4] numbers and function, the latter being also linked to a T cell exhaustion phenotype with high PD-1 expression potentially explaining limited anti-leukemic potency. In contrast to TFR-prediction, robust diagnostic prediction markers of deep molecular response to TKI response are still lacking. Such markers would potentially improve patient management by preventing over- or under-treatment and saving costs. Particularly in times when novel therapy concepts (which may enhance side effects) are tested in combination trials (e.g. ABL001 in combination with TKIs), biomarker-based response rate estimation would help to select patients at diagnosis, that are candidates for intensified treatment concepts also justifying the potential risk of so far unknown side effects. Despite many efforts during the last decades aiming for identifying novel prognostic and predictive indicators at the time of diagnosis (in addition to the well accepted risk scores SOKAL, Hasford, EUTOS and the ETLS), robust biomarkers predicting DMR to TKI therapy are still lacking. Even systems biology techniques including mRNA or proteomic profiling did not allow for the identification of a single prognostic and/or predictive factor predicting molecular response to TKI therapy. Thus, so far response-related dynamic variables are still the most relevant predictors for long-term outcome (as mirrored in the ELN or NCCN recommendations). In a large immunophenotyping study, we recently identified various immunological biomarker for molecular response to TKI [5, 6]. Most interestingly, CD62L at diagnosis was identified as powerful novel biomarker associated with basic disease-associated variables as well as deep molecular response in previously untreated CP-CML. CD62L is a homing molecule exclusively expressed on leukocytes. It directs naive and central memory T cells into lymph nodes and enables neutrophil influx to sites of inflammation. In untreated CML increased activity of the metalloproteinase TACE induces shedding of CD62L among various other TACE substrates (e.g. TNF-α, sCD40, sTNFR1 and sVCAM1) leading to reduced surface expression and increased plasma concentrations of those molecules. High TACE activity thus contributes to a pro-inflammatory leukemia environment that may explain at least in part reduced TKI responsiveness (see Figure 1). In line with this idea, the TACE substrate and classical pro-inflammatory cytokine TNF-α is a major component of an autocrine loop supporting CML stem cell survival [7]. In addition, it is conceivable that CD62L loss modifies the anti-CML immune responses, as (leukemia-antigen specific) naive and central memory T cells usually use CD62L for recirculation into secondary lymphoid organs to be primed by antigen-presenting cells. Moreover, recent data also suggested that CD62L expression is critical for CML-SC homing to the BM stem cell niche and this work already acknowledged reduced CD62L expression in the leukemic stem cell compartment [8]. The process of aberrant TACE activation in CML however remains unknown. It appears likely that in CML, the production of factors influencing TACE activity (such as TIMP-3) is changed or alternatively an enzymatically more active TACE form is released by CML cells. Future research will define the mode of TACE deregulation and its impact on CML biology including potential immune escape. Moreover, the impact of TACE inhibitors for sensitization of TACE high CML to TKI for the improvement of molecular responses will be validated in pre-clinical and clinical models.
