# Correction: Molecular response prediction in CML: novel ideas?

**DOI:** 10.18632/oncotarget.26360

**Published:** 2018-11-09

**Authors:** Dominik Wolf, Sieghart Sopper

**Affiliations:** ^1^ Department of Hematology, Oncology and Rheumatology, Center of Integrated Oncology Cologne Bonn, University Hospital of Bonn, Bonn, Germany; ^2^ Department of Hematology and Oncology, Medical University Innsbruck, Innsbruck, Austria

**This article has been corrected:** The added affiliation information is given below:

^2^ Department of Hematology and Oncology, Medical University Innsbruck, Innsbruck, Austria

Original article: Oncotarget. 2017; 8:80105-80106. https://doi.org/10.18632/oncotarget.21049

